# Diagnosis and surgical outcomes of coarctation of the aorta in pediatric patients: a retrospective study

**DOI:** 10.3389/fcvm.2023.1078038

**Published:** 2023-07-24

**Authors:** Ting Gong, Feiyan Zhang, Lingxin Feng, Xu Zhu, Dan Deng, Tingting Ran, Liling Li, Li Kong, Liqun Sun, Xiaojuan Ji

**Affiliations:** ^1^ Department of Ultrasound, Chengdu Women’s and Children’s Central Hospital, School of Medicine, University of Electronic Science and Technology of China, Chengdu, China; ^2^ Department of Ultrasound, Children’s Hospital of Chongqing Medical University, Chongqing, China; ^3^ Department of Ultrasound, The First Affiliated Hospital of Chongqing Medical and Pharmaceutical College, Chongqing, China; ^4^ School of Medical Imaging, Changsha Medical University, Changsha, China; ^5^ Division of Cardiology, Department of Pediatrics, The Hospital for Sick Children, University of Toronto, Toronto, ON, Canada; ^6^ Department of Ultrasound, Chongqing General Hospital, Chongqing, China

**Keywords:** coarctation of the aorta, congenital heart disease, transthoracic echocardiography, computed tomographic angiography, surgical outcome

## Abstract

**Background:**

Coarctation of the aorta (CoA) is a common congenital cardiovascular malformation, and improvements in the diagnostic process for surgical decision-making are important. We sought to compare the diagnostic accuracy of transthoracic echocardiography (TTE) with computed tomographic angiography (CTA) to diagnose CoA.

**Methods:**

We retrospectively reviewed 197 cases of CoA diagnosed by TTE and CTA and confirmed at surgery from July 2009 to August 2019.

**Results:**

The surgical findings confirmed that 19 patients (9.6%) had isolated CoA and 178 (90.4%) had CoA combined with other congenital cardiovascular malformations. The diagnostic accuracy of CoA by CTA was significantly higher than that of TTE (*χ*^2^ = 6.52, *p* = 0.01). In contrast, the diagnostic accuracy of TTE for associated cardiovascular malformations of CoA was significantly higher than that of CTA (*χ*^2^ = 15.36, *p* < 0.0001). Infants and young children had more preductal type of CoA, and PDA was the most frequent cardiovascular lesion associated with CoA. The pressure gradient was significantly decreased after the first operation, similar at 6 months, 1 year, and 3 years follow-ups by TTE.

**Conclusions:**

CTA is more accurate as a clinical tool for diagnosing CoA; however, TTE with color Doppler can better identify associated congenital cardiovascular malformations. Therefore, combining TTE and CTA would benefit clinical evaluation and management in patients suspected of CoA. TTE was valuable for post-operation follow-up and clinical management.

## Introduction

Coarctation of the aorta (CoA) is a cardiovascular malformation commonly described as the narrowing of the aortic isthmus between the left subclavian artery and ductus arteriosus, which has been reported to take three forms: preductal, ductal, and postductal CoA (1). CoA accounts for approximately 5%–7% of all congenital heart defects (2) with an incidence of 2.5–4 per 10,000 live births (3) and is approximately 4 times more frequent in males than females (4). CoA can be presented as a solitary defect but has been associated with other cardiac malformations such as bicuspid aortic valve, transposition of the great arteries, ventricular septal defect (VSD), and patent ductus arteriosus (PDA) (5). Despite the progress made in fetal diagnosis and treatment, prenatal screening for CoA is a diagnostic challenge. It is the most commonly missed diagnosis of fetal congenital heart disease (CHD), with less than one-third of cases detected (6, 7). More importantly, 60%–80% of newborns with CoA are not diagnosed before hospital discharge (8–11), and one study found that 27% of undiagnosed patients with CoA died at a median age of 17 days (12). Furthermore, recent studies have shown that in the presurgical era the median survival age was 31 years (13, 14).

Newborns are usually asymptomatic right after birth because of the PDA. Some infants complained manifestation of clinical symptoms: cardiogenic shock, absent/feeble femoral pulse, delayed capillary refill, feeding problems, decreased responsiveness, metabolic acidosis, mesenteric ischemia, myocardial depression, etc. (14). Unrepaired CoA leads to premature coronary artery disease, ventricular dysfunction, aortic aneurysm/dissection, and cerebral vascular disease by the third or fourth decade of life (7, 15, 16). However, those who have got surgical or transcatheter intervention, the natural history of the disease has significantly changed with most patients making it to adulthood (14). There is a need to improve diagnosis and early intervention to prevent unrepaired CoA complications. Postnatally, transthoracic echocardiography (TTE) is the gold standard imaging modality for preliminary and diagnostic screening for clinical suspicion of CoA, which involves 2D echo for structural evaluation and color Doppler for the direction of blood flow (6).

Different modalities could be used to assess CoA, including chest radiography, transthoracic echocardiography, computed tomography (CT) and computed tomographic angiography (CTA), cardiac magnetic resonance imaging (CMR), and catheter angiography (6). However, advanced modalities such as CMR and CTA confirm a suspected vascular diagnosis. Primary imaging of TTE modality for suspected CoA has given its availability, safety, and capacity to provide hemodynamic parameters. TTE can assess cardiac function and associated cardiac and valvular abnormalities. TTE is limited in evaluating extracardiac structures and collateral circulation due to poor acoustic window and operator dependence (6). It is one of the first-line imaging modalities used in the evaluation of cardiac function and disease owing to its low cost, portability, widespread availability, and lack of ionizing radiation. It is important for CoA to be diagnosed and repaired early due to its high mortality rate and the fact that even those with repaired CoA are now no longer considered benign conditions and need continuous follow-up (17). This retrospective study aimed to assess the diagnostic accuracy of TTE with CTA for the diagnosis of CoA, and the clinical management and surgical outcomes.

## Materials and methods

### Study population

**Figure 1:**
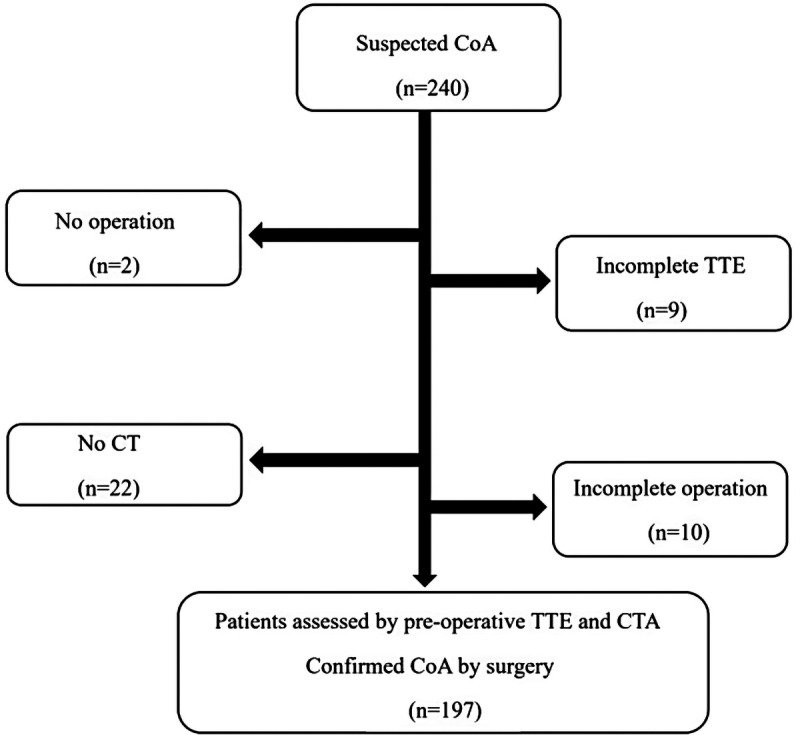
A flowchart of this retrospective cohort.

We collected the patients suspected of CoA and excluded ineligible patients (Figure 1). A total of 197 children assessed by preoperative TTE and CTA with a diagnosis of CoA confirmed at the surgery between July 2009 and August 2019 were included in this retrospective study. Overall, each patient had a preoperative diagnostic TTE and CTA and a postoperative TTE. The patients with incomplete clinical data were excluded from the research. This retrospective study was approved by the research ethics committee of the Children's Hospital of Chongqing Medical University, China.

### TTE examination

The patients were examined by TTE using a Philips IE33, GE Vivid I, and GE E9 color Doppler ultrasonic instrument with a 1–8 MHz transducer. The children less than 3 years old were orally or rectally given chloral hydrate sedative at a dose of 0.5 ml/kg. The patients underwent detailed echocardiography according to published guidelines (18). The apical four-chamber view, left ventricular (LV) long-axis view, large artery short-axis view, and suprasternal view were assessed to confirm the structures and connections of the atria, ventricles, and aorta. The suprasternal view was used to assess the aortic arch and its branches. In addition, the descending aorta was assessed to determine the location and extent of the stenosis. The location and the diameter of the narrowest part at the coarctation site were evaluated. The segment of the aortic arch was measured from the inner edge to the inner edge on two-dimensional images during systole. In addition, the maximum blood flow velocity and pressure gradient by color Doppler at the coarctation site were recorded.

### CTA examination

Before the CT examination, the guardians of the patients provided informed consent for the potential adverse reactions to contrast medium and exposure to radiation. The images were acquired with Lightspeed VCT 64-slice spiral CT (GE Healthcare, USA) and Brilliance ICT 256-slice spiral CT (Philips, Netherlands) by using the following parameters: tube voltage, 90–120 kV; automatic tube current, 60–100 mAs; pitch, 0.984; slice thickness, 5.0 mm; and slice interval, 5.0 mm. The patients were scanned in the supine position from the neck to the diaphragm. A contrast agent (Omnipaque, GE Healthcare, USA) was injected during the scan into the scalp vein or elbow vein of the patient at 300–350 mg/ml at 2–5 ml/s, dosage of 2.0 ml/kg. The children under 5 years old were sedated with contrast agent (0.5 ml/kg). The post-processing methods in the CT scanner included multiplanar reconstruction and three-dimensional volume rendering. All images were reconstructed with 1.25 mm slice thickness with a 0.625 mm slice interval. Multi-directional and multi-angle reconstruction images were used to evaluate the heart structure and origin of the blood vessels.

### Surgical management and outcome follow-up

All cases in this cohort underwent end-to-end anastomosis, end-to-side anastomosis, extended end-to-end anastomosis, extended end-to-side anastomosis, pulmonary artery patch, pericardial patch, GORETEX patch or aortic release depending on the different type of CoA and associated cardiac malformations, the aortic arch, innominate artery, left common carotid artery, left subclavian artery, and descending aorta were completely released to prevent recoarctation. A patient follow-up was required until the first discharge and checked if any patient underwent the second operation. Transcoarctation systolic pressure gradient higher than 20 mmHg was considered a recoarctation. The postoperative pressure gradient was followed up at post-operation before discharge, 6 months, 1 year, and 3 years by TTE.

### Statistical analysis

Graphpad 9.0 (Prism, USA) was used for the data analysis. Quantitative data with normal distributions were expressed as mean ± standard deviation (SD). The Chi-squared test (*χ*^2^) was used to compare the diagnostic accuracy rate of TTE and CTA, and the rates in preductal, ductal, and postductal groups. The Mann–Whitney test, paired *t*-test, and ANOVA were used in groups. A *p*-value of <0.05 was considered statistically significant.

## Results

Of the 197 children assessed, there were 129 (65.5%) males and 68 (34.5%) females, with a median age of 4.1 months (1 day–14.5 years) and a median weight of 2.5–44 kg. Among these cases, 174 (88.3%) children were under 3 years old. The symptoms that prompted a clinical investigation for these patients included dizziness, headache, hypertension, recurrent respiratory infection, shortness of breath, feeding difficulty, developmental delays, and abnormal blood pressure gradient between the upper and lower extremities.

### TTE

**Figure 2:**
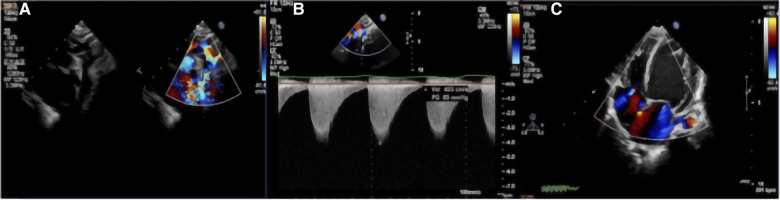
TTE with color Doppler flow imaging showed the location of the CoA and increased blood flow velocity: (**A**) indicate the location of the CoA and multicolored flow signal; and (**B**) increased blood flow velocity with a high-pressure gradient, which was estimated by a continuous-wave Doppler. (**C**) Left ventricular hypertrophy.

The two-dimensional images captured from TTE showed luminal narrowing of the aorta. Despite the different types of CoA (preductal, ductal, or postductal), a pre- and post-stenotic dilatation often developed. The color Doppler images showed an increased systolic blood flow velocity with multicolored flow signal, and a blood pressure gradient of >20 mmHg. The blood flow velocity was decreased at the distal end of the stenosis. In addition, left ventricular hypertrophy, thickened interventricular septum and left posterior ventricular wall, and decreased left ventricular systolic function were observed (Figure 2). The postoperative median inner diameter and systolic pressure gradient of the preductal, ductal, and postductal CoA were all significantly higher than the preoperative measurements (*p* < 0.0001) (Table 1). However, there were no significant differences between the three different types of CoA in terms of the internal diameter of the aorta and pressure gradient at the pre- and post-surgical TTE evaluation (*p* > 0.05).

**Table 1:** Preoperative and postoperative stenosis diameters in different types of CoA.

Types	Stenosis diameter (mm)		Pressure gradient (mmHg)	
	Preoperative	Postoperative	Preoperative	Postoperative
Preductal	3.7 (3.1–4.5)	6.0 (5.2–7.2)***	40.0 (33.8–55.5)	22.0 (17.0–31.0)***
Ductal	3.8 (3.1–4.5)	6.5 (5.3–7.6)***	45.0 (37.0–60.5)	23.0 (17.5–30.5)***
Postductal	3.9 (3.1–4.5)	6.6 (5.15–8.1)***	43.5 (33.5–53.0)	24.5 (18.8–30.3)***

*** *p* < 0.0001.

### CTA

**Figure 3:**
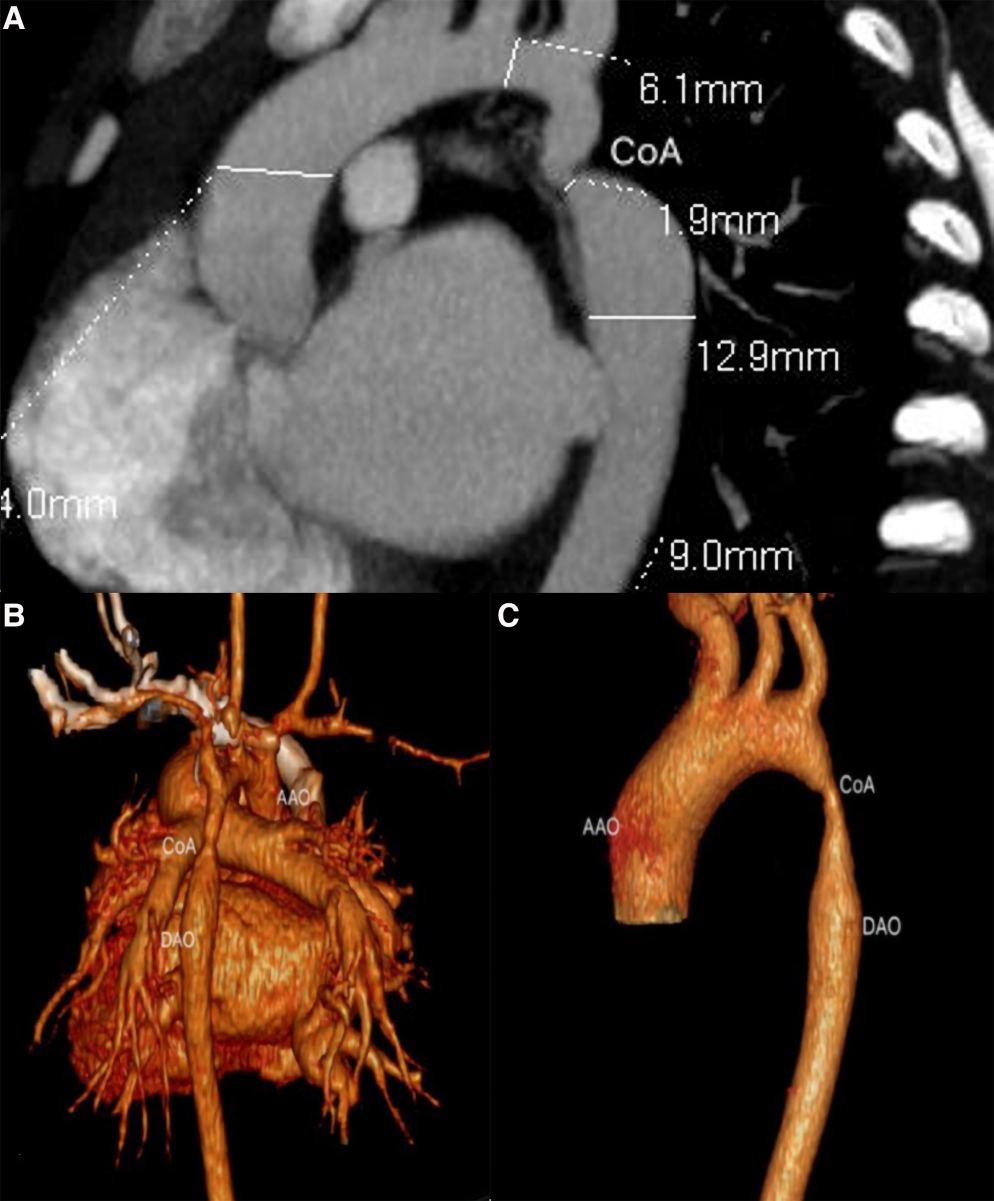
Ductal CoA by CTA. (**A**) The anatomical measurements by CTA before cardiac surgery; (**B,C**) sagittal multiplanar reconstruction showed volume rendering images with CoA.

A three-dimensional reconstruction from multiple two-dimensional CTA acquisitions established the opportunity to evaluate aortic coarctation vessel diameter, location and degree of stenosis (Figure 3), and associated vascular malformations. CTA was superior to TTE in detecting aortic arch hypoplasia and delineating great vessel branching. The reconstructed three-dimensional CTA images showed the ascending aorta, brachiocephalic artery, left common carotid artery, left subclavian artery, descending aorta, coarctation sites, and collateral circulation. In short, CTA comprehensively showed the diameter of the aortic coarctation, the morphology and anatomy of the aortic arch and the heart, and the state and number of collateral circulations before surgery.

### TTE vs. CTA diagnostic accuracy

The diagnostic accuracy of TTE and CTA were compared against the diagnosis of CoA confirmed at surgery. Of the 197 cases assessed, 184 patients (93.4%) were diagnosed correctly by TTE, nine (4.6%) were missed, and four cases (2.0%) were misdiagnosed as interrupted aortic arch (IAA) type B. In comparison, 194 children (98.5%) were diagnosed correctly by CTA, 2 cases (1.0%) were misdiagnosed as IAA, and 1 (0.5%) was missed. The diagnostic accuracy of CTA was significantly higher than TTE for CoA (*χ*^2^ = 6.52, *p* = 0.01) (Table 2). One patient with a preductal CoA was misdiagnosed in both TTE and CTA examinations. There were 468 associated cardiovascular malformations confirmed at surgery (Table 3). Among them, 434 were correctly detected by TTE. At the same time, 34 were misdiagnosed, with 4 cases misdiagnosed as other malformations: 1 patent foramen ovale (PFO), 1 atrial septal aneurysm, 1 collateral, and 1 vascular malformation (pulmonary artery origin); and 30 cases misdiagnosed from being undetected: 12 hypoplastic aortic arch (HAA), 6 collateral circulation, 3 PDA, 2 persistent left superior vena cava (PLSVC), 2 ventricular septal defect (VSD), 1 atrial septal defect (ASD), 1 aortic valve hypoplasia, 1 PFO, 1 aortic angulation, and 1 total anomalous pulmonary venous connection (TAPVC).

**Table 2:** Diagnostic accuracy of TTE and CTA compared with surgery.

	CTA vs. TTE	CTA + TTE vs. TTE	CTA + TTE vs. CTA
CoA	98.5% vs. 93.4%	99.5% vs. 93.4%	99.5% vs. 98.5%
*χ* ^2^	6.52	10.66	1.01
P	0.10	0.0011	0.31
Associated cardiac malformations	84.6% vs. 92.7%	97.6% vs. 92.7%	97.6% vs. 84.6%
*χ* ^2^	15.36	12.35	49.19
*p*-value	<0.0001	0.0004	<0.0001

**Table 3:** CoA with different cardiac malformations.

Types (*n* = 197)	*n* (%)
PDA	117 (59.0%)
VSD	116 (59.0%)
Atrial septum defect	104 (52.5%)
PFO	33 (16.8%)
Hypoplastic aortic arch	22 (11.2%)
Vascular malformation	19 (9.6%)
PLSVC	17 (8.6%)
Collateral circulation	14 (7.1%)
Valvular lesions	14 (7.1%)
Double outlet right ventricle	3 (1.5%)
Atrial septal aneurysm	2 (1.0%)
Transposition of great arteries	2 (1.0%)
Double-chambered left ventricle	2 (1.0%)
Double-chambered right ventricle	2 (1.0%)
Endocardial fibroelastosis	1 (0.5%)

In comparison, 396 associated cardiovascular malformations were correctly detected by CTA, and 72 were misdiagnosed, with 11 cases misdiagnosed as other malformations: 5 PFO, 3 PDA, 1 ASD, 1 aortic arch dysplasia, and 1 vagus subclavian artery. The undetected cases include 15 PFO, 12 VSD, 10 valvular malformations, 7 collaterals, 1 PDA, 2 TAPVC, 3 vascular malformations, 2 HAA, 1 aortic angulation, 1 endocardial elastofibrosis (EFE), 1 PLSVC, 1 ASD, 1 atrial septal aneurysms, 1 double-chambered left ventricle (DCLV), 1 double-chambered right ventricles (DCRV), and 1 double outlet right ventricle (DORV). The diagnostic accuracy of TTE and CTA for associated cardiovascular defects was 92.7% (434/468) and 84.6% (396/468), respectively, where the diagnostic accuracy of TTE was significantly higher than CTA for CoA (*χ*^2^ = 15.36, *p* < 0.0001) (Table 2).

### Surgical management and outcomes

**Figure 4:**
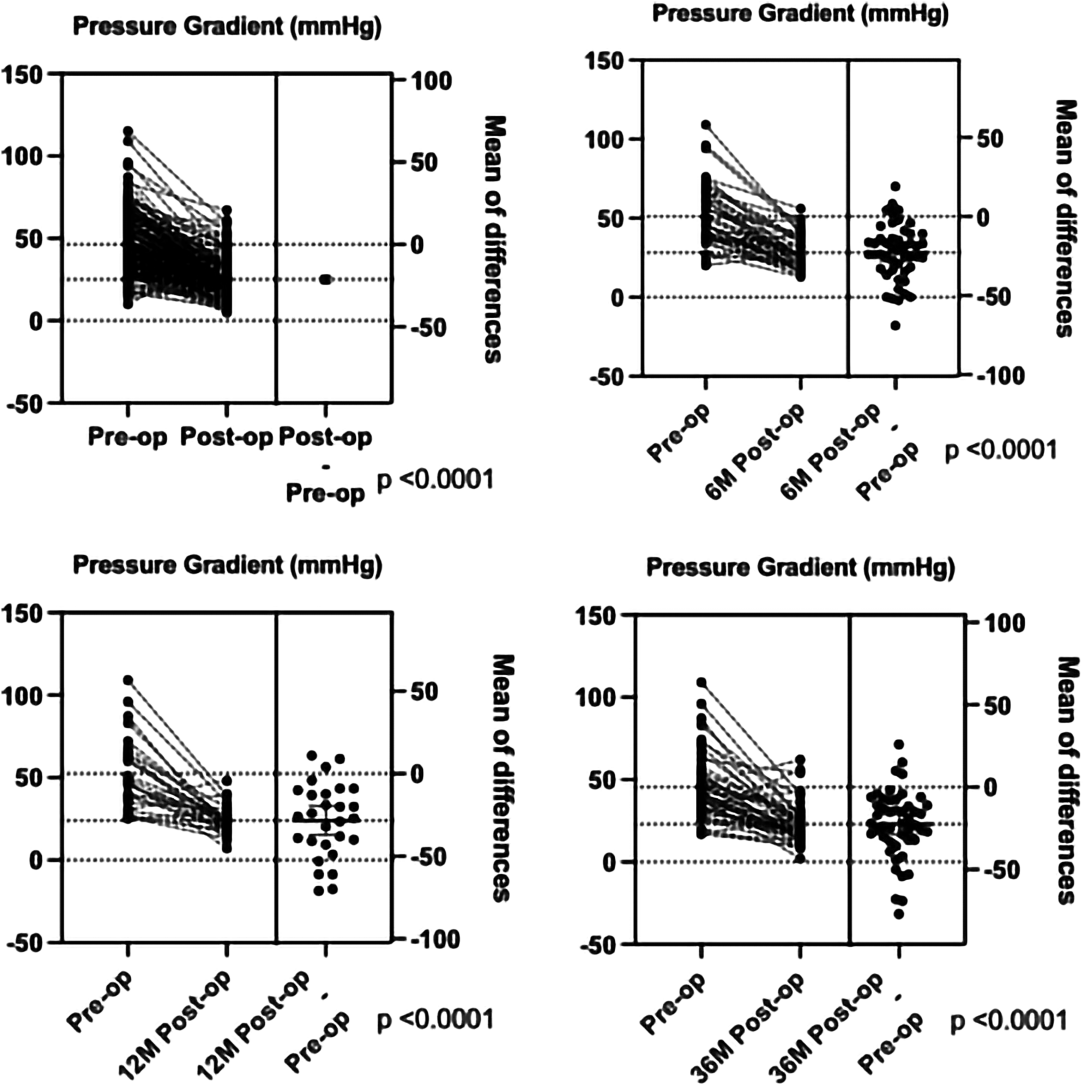
The comparison of the pressure gradient between pre-operation and post-operation before discharge, 6 months, 1 year, and 3 years by TTE.

Based on the surgical findings, three types of CoA were identified: the preductal type (*n* = 93, 47.2%), the ductal type (*n* = 86, 43.7%), and the postductal type (*n* = 18, 9.1%) (Table 4). The surgical findings confirmed that 19 patients (9.6%) had isolated CoA and 178 (90.4%) had CoA combined with other congenital cardiovascular malformations (Table 3). The top five congenital cardiovascular malformations combined with CoA were PDA (25.0%), VSD (24.8%), ASD (22.2%), PFO (7.1%), and HAA (4.7%). The surgical procedures predominantly included extended end-to-end anastomosis (*n* = 20), extended end-to-side anastomosis (*n* = 62), end-to-end anastomosis (*n* = 47), end-to-side anastomosis (*n* = 30), pulmonary autograft patch aortoplasty (*n* = 20), aortic arch release (*n* = 10), and others (*n* = 8). Among them, the surgical procedures with isolated CoA included end-to-end anastomosis (*n* = 11), end-to-side anastomosis (*n* = 4), aortic arch release (*n* = 2), extended end-to-end anastomosis (*n* = 1), and extended end-to-side anastomosis (*n* = 1) (Table 5). The surgical management of different types of non-isolated CoA is shown in Table 6. Out of the 197 cases, 191 cases (97.0%) survived after surgical repair, and in-hospital mortality occurred in six patients (3.0%). One patient died from renal failure and had a preductal CoA with severe mitral stenosis, PDA, VSD, and ASD. The other five patients died from heart failure: two patients had DORV, VSD, ASD, and PDA, one of which died during the operation while the other patient died post-surgery in addition to respiratory failure; one other patient had abnormal origin of the right pulmonary artery with postoperative pulmonary hemorrhage; one with VSD and huge ASD died as severe arrhythmia and gastrointestinal bleeding; one with preductal CoA, ASD had severe bradycardia and died with the pacemaker. The postoperative pressure gradient was significantly decreased at post-operation before discharge, 6 months, 1 year, and 3 years (Figure 4). Although the TTE systolic pressure gradient in some patients was higher than 20 mmHg, most had no clinical symptoms, and TTE indicated a normal left heart function without significant recoarctation. Therefore, no patient underwent re-operation diagnosed with recoarctation. When the pressure gradient was higher than 40 mmHg, patients received clinical consultation.

**Table 4:** Clinical management and short-term outcomes in different types of CoA.

	Preductal (93)			Ductal (86)			Postductal (18)		
	Isolated CoA	Intracardiac malformation	Extracardiac malformation	Isolated CoA	Intracardiac malformation	Extracardiac malformation	Isolated CoA	Intracardiac malformation	Extracardiac malformation
*n*	0	10	83	17	23	46	2	1	15
Age (days)	0	75 (33–126)	115 (49–281)	288 (154–1,015)	89 (46–395)	110 (39–348)	58–3,710	79	249 (50–88)
Weight (kg)	0	4.5 (4.0–5.5)	4.5 (3.7–6.6)	7.5 (6.0–12.0)	5.2 (3.9–7.8)	5.3 (4.0–8.3)	4.5–35	4.0	5.3 (3.6–11.5)
Male	0	3/9	55/84	10/17	17/23	16/46	2/2	1/18	12/18
Length of stay (days)	0	30 (25–36)	31 (22–38)	20 (14–28.5)	29 (20–38)	27 (20–34)	17–18	46	27 (19–32)
PICU stay (*n*)	0	5	49	12	18	36	1	1	11
PICU (days)	0	2 (2–2.5)	5 (3–10)**	7.5 (5.5–11)	5.5 (2–8)	6 (4–7)	1	7	7 (3–10)
CCU stay (*n*)	0	5	47	15	19	42	1	1	14
CCU (days)	0	3 (2.5–3.5)	3 (3–4)	4 (2–6)	3 (3–5)	3 (3–5)	5	4	3.5 (2–8)
Ventilation (*n*)	0	5	49	17	22	43	2	1	15
Ventilation time (h)	0	17 (15–25)	66 (21–115)*	96 (27–143)	64 (18–143)	84 (46–122)	16–24	139	70 (8–137)
Systemic circulation (*n*)	0	5	39	17	15	34	1	1	10
Systemic circulation time	0	130 (118–183)	160 (123–196)	146 (138–153)	131 (102–160)	148 (129–170)	230	155	147 (144–161)
Clamp (*n*)	0	5	43	13	15	35	1	1	10
Clamp time	0	58 (56–97)	67 (58–73)	83 (50–101)	60 (44–73)	78 (60–97)*	112	95	82 (72–104)
Complications (*n*)	0	3	38	0	8	19	0	0	7
Death (*n*)	0	0	3	0	1	1	0	0	1

*p < 0.05, **p < 0.01

**Table 5:** Surgical managements of different types of isolated CoA.

Type of cardiac surgery	Preductal	Ductal	Postductal
	Isolated CoA	Isolated CoA	Isolated CoA
*n*	0	17	2
Extended end-to-end anastomosis	0	1	0
Extended end-to-side anastomosis	0	1	0
End-to-end anastomosis	0	10	1
End-to-side anastomosis	0	3	1
Pulmonary patch aortoplasty ES	0	0	0
Aortic arch release	0	2	0
Pericardial patch	0	0	0
GORETEX patch	0	0	0
Switch	0	0	0

**Table 6:** Surgical management of different types of non-isolated CoA.

Type of Cardiac Surgery	Preductal (93)		Ductal (69)		Postductal (16)	
	Intracardiac malformation	Extracardiac malformation	Intracardiac malformation	Extracardiac malformation	Intracardiac malformation	Extracardiac malformation
*n*	10	83	23	46	1	15
Extended end-to-end anastomosis	0	13	1	5	0	0
Extended end-to-side anastomosis	5	34	6	11	0	5
End-to-end anastomosis	1	10	9	10	0	6
End-to-side anastomosis	3	9	4	9	1	0
Pulmonary patch aortoplasty ES	0	9	2	8	0	1
Aortic arch release	0	5	0	2	0	1
Pericardial patch	0	2	1	1	0	0
GORETEX patch	0	1	0	0	0	1
Switch	1	0	0	0	0	1

## Discussion

Our cohort consisted of 197 patients with a male:female ratio of 1.87:1, slightly lower than the reported literature. The pathophysiological mechanisms that underlie CoA remain poorly understood and have been described to be multifactorial. These hypotheses include the following: (1) abnormal embryogenetic development, (2) reduced vessel blood flow, and (3) aberrant duct-like arterial tissue constriction around the aortic lumen at the isthmus (6, 19). One study suggested that coarctation frequently occurs at the ductal position (20). However, another study showed that infants and young children had more of the preductal CoA type (21), which aligns with our results. Our clinical data were composed of 174 infants less than 3 years old, which suggests that preductal CoA commonly presents in infancy. This is important because if patients, particularly newborns, have severe aortic coarctation, their left ventricle cannot efficiently pump blood against the high pressure in the aorta (1), and thus, it leads to hypertrophy. CoA is often accompanied by other cardiovascular anomalies. In this study, 19 patients had isolated CoA, and 178 children had CoA combined with other congenital cardiovascular malformations, consistent with the previous data (13, 22). In the literature, bicuspid aortic valve is the most associated abnormality that affects ≤75% of all CoA cases (23). However, our results suggested that PDA was the most frequent cardiovascular lesion associated with CoA. The treatment options for CoA include surgery and endovascular intervention, including surgical, balloon angioplasty, and stent treatment (24, 25). Because of our retrospective study design, all our patients had undergone surgery based on preoperative TTE and CTA results, with 41.4% of the patients undergoing extended end-to-end anastomosis and extended end-to-side anastomosis, 39.4% of the patients with end-to-end anastomosis and end-to-side anastomosis, 13.1% of the patients with the patch, including patch aortoplasty, pericardial patch and GORETEX patch. The surgical decision-making depended on the type of CoA and associated cardiac malformations.

In this group, 184 cases were correctly diagnosed by TTE with nine cases undiagnosed and the remaining four cases misdiagnosed as IAA. The limitations of TTE included poor acoustic window, narrow field of view, and operator dependence. Two CoA cases with PDA and VSD were misdiagnosed, which we suggest was because the forward blood flow velocity in the descending aorta did not change significantly and thus no multicolored flow signal was detected by color Doppler. Vergales et al. suggested that a PDA in addition to CoA might affect the physiologic obstruction in neonates (26). Therefore, coarctation in newborns might not be present until the ductal tissues insert completely. In CoA with VSD, the left ventricular end-diastolic pressure is increased by the ventricular preloading, and it is shown as cardiac hypertrophy and left heart dysfunction, which is caused by the progressive of pulmonary venous and arterial hypertension (27). Severe CoA can be distinguished from IAA if there is a long stenotic section. The patients with IAA exhibit blood flow interruption between the ascending aorta and the descending aorta. Thus, the blood that flows into the descending aorta is solely supplied by the PDA. Most diagnoses of IAA are accompanied by ascending aortic arch dystrophy and moderate to severe pulmonary hypertension (28). However, this is not usually associated with pulmonary hypertension unless other cardiovascular abnormalities exist (29). Several imaging methods have been used to detect CoA. Conventional angiography was regarded as the gold standard for clinical assessment of the aorta. However, it is invasive and requires considerable radiation exposure (30). In addition, magnetic resonance imaging is a common tool for vascular diseases, but is limited by its high cost, long examination times, and patients with claustrophobia (31). TTE often serves as the first-line imaging tool for assessing cardiovascular function. It has been widely used for diagnosing CoA and other accompanying cardiovascular lesions due to its low cost and radiation-free properties (32).

CTA is now considered a more reliable technique than TTE for the diagnosis of aortic diseases, because it allows better visualization, especially in the detection of extracardiac-vascular abnormalities, with short acquisition time and high spatial resolution (4). With the recent advances in modern scanning techniques, the overall radiation burden required for CTA has significantly reduced, which means that this examination is relatively safe for infants and young children. However, considering that young patients require lifelong follow-up, radiologists should be careful with the use of ionizing radiation in serial examinations. In the current study, the accuracy rate of CTA for the diagnosis of CoA was significantly higher than that of TTE (*p* = 0.01). The multi-directional and multi-angle reconstructed images from CTA accurately detected the origin and morphology of the great vessels, and their relationship with the heart, which might compensate for the limitations of TTE. Although CTA was superior to TTE when assessing extracardiac-vascular anomalies, the associated cardiovascular lesions remain difficult to detect by CTA alone. TTE failed to diagnose 34 associated cardiovascular malformations, which included 12 HAA, 10 vascular malformations, 3 PDA, 2 VSD, 1 ASD, 1 aortic valve hypoplasia, 2 PFO, and 1 atrial septal aneurysm, 1 collateral and 1 vascular malformation (pulmonary artery origin).

In comparison, CTA misdiagnosed 72 associated cardiovascular malformations, which included 20 PFO, 19 vascular-related malformations, 14 VSD, 13 valvular lesions, 1 EFE, 1 PLSVC, 1 atrial septal aneurysm, 1 DCLV, 1 DCRV, and 1 DORV. These data suggest that TTE was better than CTA in detecting associated cardiovascular lesions, presumably because TTE provides hemodynamic information. In addition, TTE combined with color Doppler can evaluate blood shunting, valvular anomalies, and other intracardiac structures.

Furthermore, TTE has been widely used in postoperative follow-up of patients with repaired CoA for recoarctation. Currently, we use TTE to screen patients with suspected CoA by anatomic structural abnormality, the increased flow velocity of the descending aorta, and assess the intracardiac malformations, using CTA to confirm the diagnosis of CoA, and then managing the surgical plans. The in-hospital mortality rate was 4.9% in this group, which is higher than a large cohort that analyzed 2,424 CoA cases from 43 centers. The main reason for the deaths was attributed to congenital heart disease or other complications. Regarding long-term survival, the outcomes of patients with repaired CoA in infancy were excellent, but they had a small ongoing mortality risk in the early childhood (24). As recoarctation and high blood pressure may occur after repair (22, 33), it is important to continue long-term clinical and cardiovascular imaging follow-up in this population. We showed a significantly decreased pressure gradient after the operation, similar at 6 months, 1 year, and 3 years follow-ups. Although some patients still had systolic pressure gradient higher than 20 mmHg, no patient underwent the second operation with significant recoarctation in our follow-up. We recommend that if patients have no significant clinical symptoms and have normal left heart function, then they can undergo dynamic observation by TTE.

The median age of death is 31 years if 60%–80% of newborns are undiagnosed. Increased left ventricular afterload can cause the potential downstream effects (34, 35). CoA represents a form of pressure afterload that affects the LV. Correction of CoA before irreversible LV dysfunction is vital, which is more crucial if associated with other cardiovascular malformations. LV longitudinal strain is decreased, and LV maximum torsion is elevated in children with moderate LV pressure load secondary to LV obstructive lesions such as mild CoA, which changes rapidly after a successful interventional treatment resulting in LV maximum torsion reduction (36). In another study, the ejection fraction remained normal, but a decrease in strain was measured. Distinct left ventricular outflow tract obstruction (LVOTO) demonstrates different strain values. It is important for an early heart failure treatment to be performed in cases of LV aberrations before the onset of functional abnormalities (37). However, one study showed that non-neonatal CoA repair may be more beneficial than neonatal CoA repair in terms of LV systolic dysfunction recovery, but this may be confounded as neonates intervened during the neonatal period may have more severe CoA, which leads to circulatory problems and early intervention (38). When the PDA closes after birth, severe CoA will lead to aortic obstruction with hypoperfusion of the lower body, renal dysfunction, and metabolic acidosis. Increased afterload of the LV may lead to LV failure. Prostaglandin E is needed to keep the PDA open for lower body perfusion. Milder cases of CoA are important because hypertension and compensatory LV hypertrophy due to increased LV afterload may occur later in life (6). Limited restrictions are indicated for patients with high blood pressure, recoarctation, and aorta dilatation. There is an excellent survival but with a small ongoing mortality risk into early adulthood (39), and lifelong risk of developing aortic aneurysm, hypertension, and cerebral aneurysms. Therefore, patients diagnosed with CoA should seek lifelong follow-up with a cardiologist. Lifelong regular cardiology evaluations are important to monitor for later complications (24). Adequate and timely diagnosis of CoA is crucial for a good prognosis, as early treatment is associated with lower risks of long-term morbidity and mortality (6). Overall, our retrospective study presented the diagnosis and surgical management and outcome follow-up in the large Chinese population and summarized the surgical prognosis of CoA.

### Limitation

This study was limited by its retrospective design where some patients had incomplete clinical data, and not all patients had genetic testing performed. Furthermore, only the raw diameter of the CoA was recorded as there is no adequate and available *Z*-score COA calculator for the Chinese population when quantifying the TTE results. We only assessed patients who underwent surgical intervention and not balloon or stent catheterization. Since this was surgery, these cases were probably more extreme compared with those who ended with a catheterization treatment. For the pressure gradient follow-ups by echocardiography, as this was a retrospective study, the follow-up rate of the surgical outcome was lower than expected at 6 months, 1 year, and 3 years.

## Conclusions

This study demonstrates that the diagnostic accuracy of CTA for CoA was higher than that of TTE, and TTE was superior to CTA when detecting other cardiovascular lesions. The combined use of CTA and TTE may improve the preoperative diagnostic accuracy of CoA and associated cardiovascular malformations, respectively, providing a more comprehensive assessment for the clinical management and surgical decision-making. TTE was beneficial for post-operation follow-up and clinical management.

## Data Availability

The original contributions presented in the study are included in the article, further inquiries can be directed to the corresponding authors.
